# How to objectively determine the color of beer?

**DOI:** 10.1007/s13197-020-04237-4

**Published:** 2020-01-09

**Authors:** Dániel Koren, Beáta Hegyesné Vecseri, Gabriella Kun-Farkas, Ágnes Urbin, Ákos Nyitrai, László Sipos

**Affiliations:** 1 Department of Brewing and Distilling, Faculty of Food Science, Szent István University, 45 Ménesi út, Budapest, 1118 Hungary; 2 Department of Mechatronics, Optics and Mechanical Engineering Informatics, Faculty of Mechanical Engineering, Budapest University of Technology and Economics, 3. Műegyetem út, Budapest, 1111 Hungary; 3 Department of Postharvest Science and Sensory Evaluation, Faculty of Food Science, Szent István University, 29 Villányi út, Budapest, 1118 Hungary

**Keywords:** Beer, Transmission spectra, Color, CIE 1976 L*a*b* color space, Color difference

## Abstract

Beer color is an important sensory attribute, the first one that the consumer observes. There are two standard methods accepted for determining the color of these products, one related to the European Brewery Convention (EBC) and the other is the Standard Reference Method (SRM). Both methods are based on absorbance, but in case of the more and more popular fruit beers these methods give false result since these products appear in varied colors and have different spectra than regular beers. In this study 39 different types of beers were investigated, including fruit beers and beer based mixed drinks to compare their color in CIE 1976 L*a*b* color space, absorption-based colors and transmission spectra. ΔE*_ab_ values of products with less than 5% EBC difference ranged from 4.5 to 17.4. There were magnitude differences in the transmission spectra of these products, fruit beers showed different tendencies due to the added fruit or fruit juice. The highest ΔE*_ab_ value belonged to two traditional Weissbiers. Absorption-based methods are not able in many cases to differentiate between products which have nearly the same EBC or SRM color but visually are different. A multi-wavelength method would be reasonable to be developed for more objective and accurate beer color determination.

**Electronic supplementary material:**

The online version of this article (10.1007/s13197-020-04237-4) contains supplementary material, which is available to authorized users.

## Introduction

Brewing and beer consumption have an ancient tradition. The color of these products is an important sensory attribute, as it has to be true to the type of the beer and this is the first property what the consumer observes. The appearance of a product, including the reproducible foam and color, is a key quality factor.

Historically there was a need to compare the color of beers around the world that is why Lovibond method was developed, where so called comparator discs and standard illuminants were used to determine the color of the beer (Lovibond 1897). This method was subjective because it highly depended on the vision of the examiner, furthermore the ageing of the discs and the incorrect storage could cause some color shifts (Sharpe et al. 1992). Nowadays Lovibond is still used by some malters and brewers.

However there were some recommendations for multi-wavelength measurements, for instance Brandon (1957) suggested to use the ratio of absorbance measured at 460 nm and 560 nm, Nyborg and Trolle (1948) proposed the use of 430 nm and 530 nm, in the middle of the twentieth century single wavelength spectrophotometric methods were introduced. The ASBC (American Society of Brewing Chemists) and the EBC (European Brewery Convention) standardized λ = 430 nm as the measuring wavelength, because pale beers showed most variation at this wavelength. In the UK λ = 530 nm was used, because amber ales, prevalent in this region, showed most variation at this wavelength. Later λ = 430 nm was adapted in the UK as well. Since then there are two main standardized methods for the measuring of beer color. One is the Standard Reference Method (SRM) developed by the ASBC and the other is the EBC method (Hughes and Baxter 2007). These two methods differentiate beers based on their absorbance, but there are products, which have the same absorbance at λ = 430 nm but they are visually different, e.g. reddish and brown beers (Smedley 1995).

Human eye has three types of cone receptors designated as red, green and blue. These receptors exist in unequal quantities, that is why color sensitivity is determined by genetic differences in humans (Shellhammer 2009). Different color spaces were developed to model the visualization of human eye like CIE *XYZ*, CIE L*a*b*, CIE L*u*v*. They are showing different distribution as CIE *XYZ* determines the color on a three-dimensional color space based on the CIE color matching functions while CIE L*a*b* is based on opponent color theory, L* shows the brightness, the position between light and dark, a* is red versus green and b* is yellow versus blue (Bello-Cerezo et al. 2016).

The biggest problem is that color measuring methods based on absorbance were developed decades ago for traditional beers, but lately with the revolution of craft brewing and with the broadening of the palette of the international brewing companies’ fruit beers and beer-based mixed drinks are getting more popular. There is only limited information about them and about their comparison to traditional products. They are represented in many different colors and traditional color measuring methods like SRM or EBC may give false results in their case as they have other coloring components besides caramelization, pyrolysis and Maillard reaction products (Shellhammer and Bamforth 2008).

Beer-based mixed drinks are alcohol-free or low-alcohol-content beverages mainly produced by big brewing industries. These are made by mixing approximately 50% fruit juice and 50% pale beer. Fruit beers are mainly produced by smaller breweries (craft breweries) except for some Belgian examples. In case of these products fruit juice, concentrate or puree is usually added after the main fermentation for ageing or lagering, but in a remarkably less amount as in case of beer-based mixed drinks. In beer-based mixed drinks the fruit juice is the dominating flavor while in case of fruit beers according to the Beer Judge Certification Program created by Strong and England (2015): a harmonious marriage of fruit and beer, but still recognizable as a beer. The fruit character should be evident but in balance with the beer, not so forward as to suggest an artificial product, the flavor of the fruit must not dominate the basic beer type”.

The color of the final product is mainly due to the different raw materials used during the brewing process. It is primarily depending on the grains and the processes, mainly kilning or roasting, these grains have undergone (Davies 2016). The secondary contributor is the oxidation of polyphenols, originating from malt and hops, during the storage and ageing. The main phenolic components which contribute to beer color changes due to oxidation are flavan-3-ol monomers and proanthocyanidin oligomers (Aron and Shellhammer 2010).

Beer-based mixed drinks and fruit beers contain various coloring components dissolved from fruits which have influence on the results of absorbance based methods (e.g. the main coloring compounds of blackcurrant and sour cherry are anthocyanins while lemon and grapefruit contains naringin, hesperidin and eriocitrin) (Damar and Ekşi 2012; Mattila et al. 2011; Peterson et al. 2006).

As we lack information, in this preliminary study our aims were to compare the visible spectra, the EBC color and the tristimulus values calculated from the results of visible spectral analysis of the products showing less than 5% EBC color difference.

## Materials and methods

### Beer samples

39 Different beers were purchased which are available in Hungarian retail. Beers were classified based on the Beer Style Guidelines of the Beer Judge Certification Program (Strong and England 2015). We investigated three Alcohol-free pale lagers, three Alcohol-free beer-based mixed drinks, three Beer-based mixed drinks, two Strong pale lager, nine European pale lagers, two Czech pilsners, one American adjunct lager, two Schwarzbier, one Stout, one Irish stout, one Altbier, four Weissbier (unfiltered wheat beer), one International amber lager, one Belgian strong pale ale, one Irish red ale, one Dunkles bock and three Specialty fruit beers. With this data selection we aimed to involve many types of beer. Our samples are presented in Table 1. Samples were homogenized and filtered through Whatman MN-615 filter paper prior to analysis.

**Table 1:** European Brewery Convention (EBC) and Commission Internationale de l’Éclairage (CIE) L*a*b* values of the investigated samples

Sample No	EBC	CIE		
		L*	a*	b*
*Alcohol-free pale lager*				
1	7.4	95	− 2	21
2	8.2	93	− 2	22
3	8.2	94	− 3	20
*Alcohol-free beer-based mixed drink*				
4^a^	19.6	55	56	19
5^b^	10.0	86	0	20
6^c^	7.0	89	4	18
*Beer-based mixed drink*				
7^b^	5.2	92	− 1	11
8^c^	7.6	88	5	18
9^a^	15.4	63	47	15
*Strong pale lager*				
10	13.6	90	− 1	34
11	12.6	86	− 1	27
*European pale lager*				
12	8.2	95	− 3	24
13	9.1	93	− 2	24
14	8.0	94	− 3	23
15	9.0	93	− 2	25
16	6.3	95	− 2	18
17	6.2	96	− 3	18
18	7.0	94	− 2	19
19	8.1	95	− 3	23
20	8.6	93	− 2	24
*Czech pilsner*				
21	13.1	92	− 3	34
22	13.1	92	− 3	35
*American adjunct lager*				
23	6.0	96	− 3	18
*International Amber Lager*				
24	33.0	76	8	55
*Schwarzbier*				
25	81.3	47	28	2
26	93.0	40	32	8
*Altbier*				
27	71.8	58	24	49
*Belgian strong pale ale*				
28	10.3	93	− 3	29
*Weissbier*				
29	14.1	84	0	30
30	26.5	73	4	46
31	25.9	62	4	32
32	14.4	88	− 1	35
*Stout*				
33	84.9	43	28	10
*Irish red ale*				
34	34.6	70	11	51
*Irish stout*				
35	95.2	35	32	6
*Dunkles bock*				
36	96.3	34	36	5
*Fruit beer*				
37^d^	42.4	55	38	30
38^d^	53.6	51	36	29
39^d^	48.5	48	46	26

^a^Made with added sour cherry juice

^b^Made with added lemon juice

^c^Made with added grapefruit juice

^d^Aged with sour cherry

### EBC values

EBC values were determined according to the standard Analytica-EBC color measuring method (European Brewery Convention 1975). The absorbance was determined in 1 cm UV–Vis cuvettes at 430 nm by a Hach Lange DR6000 UV–Vis spectrophotometer in triplicates. The absorbances then were multiplied by 25.

### Transmission spectra

Transmission spectra was determined by a Hach Lange DR6000 UV–Vis spectrophotometer through the whole visible spectra from 380 to 780 nm with 10 nm steps.

### Calculation of tristimulus values from transmission spectra

Tristimulus values and chromaticity coordinates of the samples were calculated from transmission spectra as defined in the CIE 1931 standard colorimetric system based on the description of Commission Internationale de l'Éclairage (2004) according to the following equations

1 $$ X = k\int {\phi \left( \lambda \right){\bar{x}}\left( \lambda \right)d\lambda } $$

2 $$ Y = k\int {\phi \left( \lambda \right){\bar{y}}\left( \lambda \right)d\lambda } $$

3 $$ Z = k\int {\phi \left( \lambda \right){\bar{z}}\left( \lambda \right)d\lambda } $$

4 $$ x = X/\left( {X + Y + Z} \right) $$

5 $$ y = X/\left( {X + Y + Z} \right) $$

where *X*, *Y* and *Z* are the tristimulus values, ${\bar{x}}$ (*λ*), ${\bar{y}}$ (*λ*) and ${\bar{z}}$ (*λ*) are the CIE color matching functions, *ϕ *(*λ*) is the relative color stimulus function, *k* is a constant for normalization, *x* and *y* are the cromaticity coordinates.

The relative color stimulus function was defined as the product of the measured transmission spectra and the spectral emission of the reference illuminant that was in our case the D65 light source.

### Calculation of ΔE*_ab_ color difference

Derived from the tristimulus values L*, a*, b* coordinates and ΔE*_ab_ color differences between pairs of color samples were calculated according to Commission Internationale de l'Eclairage (2004) as the Euclidean distance between them in the CIE 1976 L*a*b* color space. Calculations followed the equations below:

6 $$ L^{*} = 116\left( {Y/Y_{n} } \right)^{3} - 16 $$

7 $$ a^{*} = 500\left[ {\left( {X/X_{n} } \right)^{3} - \left( {Y/Y_{n} } \right)^{3} } \right] $$

8 $$ b^{*} = 200\left[ {\left( {Y/Y_{n} } \right)^{3} - \left( {Z/Z_{n} } \right)^{3} } \right] $$

where *X*, *Y* and *Z* are the tristimulus values of the sample and *X*_*n*_, *Y*_*n*_, and *Z*_*n*_, are the tristimulus values of the reference light source.

Color difference between sample A and sample B was calculated equation below:

9 $$ \Delta E_{{{\text{ab}}}}^{*} = \left( {\left( {L_{A}^{*} - L_{B}^{*} } \right)^{{2}} + \left( {a_{A}^{*} - a_{B}^{*} } \right)^{{2}} + \left( {b_{A}^{*} - b_{B}^{*} } \right)^{{2}} } \right)^{{{1}/{2}}} $$

Spectral distribution of the D65 illuminant as well as the ${\bar{x}}$(*λ*), ${\bar{y}}$(*λ*) and ${\bar{z}}$(*λ*) color matching functions are available in CIE Lab Color Space_CIE deltaEab Color Difference.xlsx as ESM that can also be used for calculating L*, a* and b* coordinates of two samples and the ΔE*_ab_ color difference between them. Data should be inserted to grey cells while green cells denote results and white cells contain necessary data and calculations.

## Results and discussion

### EBC and L*a*b* color of beers

Low EBC values mean pale beer, higher EBC values describe darker beers. In case of L*a*b* the L* value can be between 0 and 100, the higher the L*, the lighter the sample. The a* and b* values can be between − 100 and + 100. The smaller a* means green, the higher red color, the smaller b* means blue, the higher yellow color.

As it can be seen on Table 1, alcohol-free pale lagers, European pale lagers and the American adjunct lager have the lowest EBC values and the highest L* values which mean that these are the palest samples as they do not contain or contain a very low amount of special malts, which can contribute to their color.

Samples containing fruit vary in EBC and L*a*b* values, which is due to the different fruits used for their production. The ones containing sour cherry juice (sample 4, 9, 37, 38, 39) have lower L* values and high a* values, which mean that they have a darker reddish color. This is due to the anthocyanins, which are the polyphenols responsible for the red color of fruit skin and flesh (Wojdyło et al. 2014). Ones containing lemon or grapefruit juice (sample 5, 6, 7, 8) have low EBC values and similar L*a*b* values to pale lagers. In case of grapefruit the distinctive color is due to lycopene, an unusual carotene in citrus fruits (Lado et al. 2015).

Czech pilsners have higher EBC values than European pale lagers which would mean that they are darker, according to the traditional color measuring method, but their L* values are similar, which mean that they are not darker, only more yellowish than European pale lagers as their b* values are higher. These agree with the results of Olšovská et al. (2014) who observed that Czech beers have higher color than other European lagers which is due to the decoction mashing technology traditionally applied for Czech lagers.

Dark beers as Schwarzbier, Stout, Irish stout and Dunkles bock have higher EBC, lower L* and higher a* values than pale beers, it is due to the use of coloring malts which were kilned, roasted at higher temperature where Maillard reaction products are formed (Hellwig et al. 2016). It would be expected that international amber lager does have high a* value which refers to reddish color but in contrast its b* value is higher. It does not contain roasted malt, but it contains caramel malt which gives its characteristic color. Altbier and Irish red ale have similarly high b* values as International amber lager has, furthermore their a* values are lower than of dark beers, which is interesting because they are visually reddish. In their case these three parameters (L*a*b*) separately cannot describe their color, they must be taken into consideration together.

Weissbier samples show values as expected, they are between pale and dark beers in darkness according to their L* value, have low a* values and high b* values, which means that they are deep yellow in color which corresponds to reality. Their color is due to the wheat malt and usually a small amount of caramel malt is also used as a raw material.

### Differences of color determining methods

**Fig. 1:**
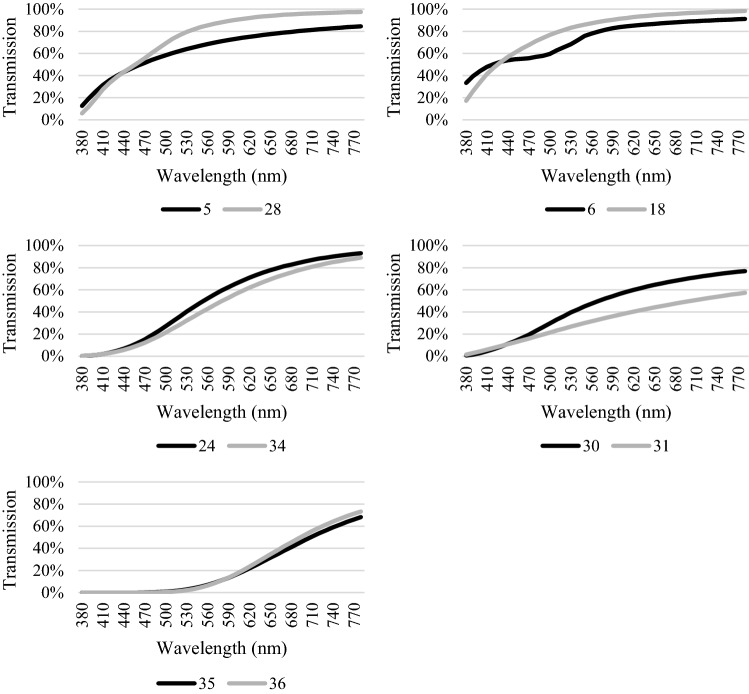
Transmission spectra of beers having less than 5% EBC color difference

The EBC and absolute L*a*b* differences (ΔE*_ab_) of the samples are shown on Table 2, while their transmission spectra are shown on Fig. 1. According to Zhu et al. (2013), if ΔE*_ab_ ≤ 1.5 it means nearly no difference in visual inspection, if ΔE*_ab_ ≥ 1.5 samples are slightly different, if ΔE*_ab_ ≥ 3.0 there is some difference and if ΔE*_ab_ ≥ 6.0, it means there is significant difference between the color of the samples.

**Table 2:** European Brewery Convention (EBC) and ΔE*_ab_ color differences (ΔE*_ab_) of beers

Beer category (sample number)	EBC difference^a^	ΔE*_ab_ difference	Visual sense difference (according to Zhu et al. 2013)
Weissbier (30) Weissbier (31)	0.6	17.4	Significant difference
Alcohol-free beer based mixed drink with lemon juice (5) Belgian strong pale ale (28)	0.3	12.6	Significant difference
Alcohol-free beer-based mixed drink with grapefruit juice (6) European pale lager (18)	0.1	7.7	Significant difference
International amber lager (24) Irish red ale (34)	1.6	7.3	Significant difference
Irish stout (35) Dunkles bock (36)	1.1	4.5	Some difference

^a^EBC differences ≤ 5%

Two Weissbier-s No. 30 and 31 have an EBC color difference of 0.6 which means less than 5% difference, on the other hand their ΔE*_ab_ is the highest among the samples. Their ΔE*_ab_ is 17.4 and their transmission spectra is very different despite of that they are the same beer type brewed from similar ingredients and with similar technology. According to their L*a*b* values, sample 31 is darker and less yellow. This difference can be explained by the different bottling and storage conditions in the supermarkets of these two products as beer color can increase through storage, especially in the presence of oxygen and at higher temperature due to the oxidation of polyphenols which can cause color shift over time (Collin et al. 2013).

The EBC difference of sample No. 5 (alcohol-free beer based mixed drink with lemon juice) and No. 28 (Belgian strong pale ale), is 0.3. The ΔE*_ab_ value is 12.6. It means that there is significant difference visually between the products. Sample 5 is an alcohol-free beer mixed with lemon juice while sample 28 is a Belgian ale produced from traditional ingredients such as water, malt and hops. Based on their L*a*b* values, sample No. 5 is darker, slightly more reddish and less yellow. This is due to the discoloration of citrus juices during storage caused by nonenzymatic browning. According to Lee and Chen (1998) this browning pigment formation in citrus juices cause a darker and less yellow color. In addition to the visual differences between the two samples, it is important to note that different chemical changes may occur during the storage of each product made from different ingredients.

Sample No. 6, an Alcohol-free beer-based mixed drink with grapefruit juice, compared to sample No. 18, a European pale lager, show lower L* and higher a* values, which mean that it is slightly darker and more red. Their b* values are similar. The ΔE*_ab_ is 7.7 (visually significantly different), however their EBC color is the same. Their transmission spectra show different tendency between ~ 420 and 560 nm. The basic beer types are similar (both are lagers), the difference is due to the added grapefruit juice to sample 6 as carotenoids, the main contributors to the color of grapefruit, have their absorption maximum between ~ 420 and 520 nm (Hempel et al. 2016). This can be clearly seen in Fig. 1, the transmission spectra of sample 6 shows a valley in this region, which would be a hill in absorption spectra.

Comparing sample 24 (International amber lager) to 34 (Irish red ale) and 35 (Irish stout) to 36 (Dunkles bock) there are no big differences in their transmission spectra, they show similar tendencies, there are small differences in their EBC color but according to their ΔE*_ab_ values, sample 24 and 34 have significant difference visually (ΔE*_ab_ = 7.3), and in case of sample 35 and 36 there is some difference between them (ΔE*_ab_ = 4.5). Although these pairs are similar beer types, the traditional method is not able to distinguish their color.

From these results, it can be clearly seen that the traditional method of color measurement is in many respects incapable of objectively determining the color of specialty beer products that are becoming increasingly popular today. This is due, among other things, to the different ingredients used in brewing (such as fruits). Since beer-based mixed drinks are generally low-alcohol or non-alcoholic, we should not forget the role of alcohol, which tends to react with free radicals among other antioxidants in traditional alcoholic products, thereby protecting the product's stability, including color (Irwin et al. 1991).

## Conclusion

Color coordinates defined in the CIE 1976 L*a*b* color space parameters calculated from transmission spectra measured in the whole visible wavelength-range can differentiate beers more objectively than methods based on absorbance. In case of Fruit beers and Beer-based mixed drinks the traditional color measuring methods cannot differentiate between products very well, which are visually different, because these products have different absorption or transmission spectra than traditional beers due to the different raw materials which contain various coloring compounds. On the other hand, based on our results, out of the five beer pairs with less than 5% EBC color difference, three pairs were traditional beer types. Furthermore, we have observed the highest visual difference in case of two Weissbiers with less than 5% EBC color difference. The production technology and recipe of these beers are very similar as they are the same beer type. It underlines that one wavelength measurement is not enough to describe the accurate color of a product, even if it is a traditional beer type. As there are more and more products available with fruit content, other additives, special raw materials and made from plenty different types of malts (e. g. craft beers), it would be reasonable to develop a standard method using color coordinates and color difference defined in the CIE 1976 L*a*b* color space based on transmission spectra measured in the whole visible wavelength-range to determine beer color more accurately and objectively.

## Electronic supplementary material

Below is the link to the electronic supplementary material.

Supplementary file1 (XLSX 27 kb)
